# Cesarean section of a patient with combined severe mitral and aortic stenosis: a case report

**DOI:** 10.1097/MS9.0000000000000291

**Published:** 2023-03-14

**Authors:** Haris Sheikh, Khalid Samad, Akbar A. Mistry

**Affiliations:** Department of Anesthesiology, The Aga Khan University Hospital, Karachi, Pakistan

**Keywords:** aortic stenosis, cesarean section, intrauterine growth restriction, mitral stenosis, valvular heart disease

## Abstract

Cardiovascular diseases during pregnancy are rare but account for complications that pose risks to the mother as well as the child. In patients with fixed cardiac output due to stenotic valvular lesion(s), the physiological changes during pregnancy carry high risk of morbidity and mortality.

**Case Presentation:**

Our patient was diagnosed with severe mitral and aortic stenosis at her first antenatal visit at 24 weeks of gestation. She was also diagnosed with intrauterine growth restriction and was therefore planned to be operated on at a gestational age of 34 weeks. After careful selection of monitoring and anesthetic regime, the patient was managed without any intraoperative or postoperative complications.

**Clinical Discussion:**

This case reports how the anesthetists, obstetricians, and cardiac surgeons devised a well-designed plan to operate on a patient with a relatively rare disease manifestation. Our patient had coexisting severe stenotic lesions of both mitral and aortic valves and posed a clinical dilemma regarding the choice of anesthesia and perioperative management. Regardless of the anesthetic technique, goals for a patient with the combined valvular disease include maintenance of adequate preload, systemic vascular resistance, cardiac contractility, sinus rhythm and avoidance of tachycardia, bradycardia, aortocaval compression, and anesthetic or surgery-induced hemodynamic changes.

**Conclusion:**

The course of management would give clinicians an idea of how to manage a patient with combined stenotic valvular lesions for cesarean section, ensuring a smooth course and a safe postoperative period.

## Introduction

Highlights

- Valvular heart disease in pregnancy is relatively common when considering comorbidities; however, combined severe mitral and aortic stenosis is a rare occurrence.
- Due to intrauterine growth restriction, the patient had to undergo a cesarean section.
- Anesthetic management poses a clinical dilemma as data is scarce, especially about the choice of anesthesia (regional vs. general anesthesia).
- Our patient’s disease was severe enough to warrant an emergency sternotomy and valve replacement if required; hence a cardiac surgeon remained scrubbed throughout the surgery.
- Perioperative management of this patient revolved around a multidisciplinary approach devised by obstetrics, cardiology, cardiac surgery, and anesthesia teams.

Anesthetic management of pregnancies with cardiac disease for labor and delivery, or cesarean section, is a challenging task. Among cardiac causes, valvular heart diseases, aortic dissection, congenital heart diseases, and cardiomyopathy are the frequently encountered etiologies^1^. Approximately 0.2–3% of pregnancies are complicated by cardiac disease. Although valvular heart diseases account for the majority of cardiac diseases complicating pregnancies, combined valvular diseases are less frequently encountered, with combined stenotic lesions being an extremely rare occurrence^2^.

We report successful anesthetic management of a patient with severe mitral and aortic stenosis presenting for cesarean section. Written and informed consent was obtained from the patient’s next of kin for publication of this report. The work has been reported in line with SCARE (Surgical CAse REport) criteria.^3^

## Case presentation

Our patient was a 34-year female, gravida 2, para 1+0, at the 34th week of gestation, presenting for elective lower segment cesarean section (LSCS) due to previous LSCS and Intrauterine Growth Restriction (IUGR) of the fetus. She had no other comorbidities, and her previous pregnancy was uneventful. She belonged to a remote village, and as such, her first antenatal visit occurred at 24 weeks of pregnancy, where she also complained of shortness of breath on minimal exertion. Her routine examination was unremarkable except for a systolic ejection murmur. She was subsequently advised an electrocardiogram (ECG) and echocardiogram apart from a routine blood workup.

Her blood work, including complete blood count, blood urea nitrogen, creatinine, electrolytes, and coagulation profile came back normal. ECG showed normal sinus rhythm at 80 beats per minute. Her echocardiogram showed an ejection fraction of 55–60% with normal biventricular systolic function, severely dilated left atrium, and mildly dilated left ventricle. The aortic valve was thickened, calcified, and severely stenosed with a valve area of 0.70 cm^2^ with peak and mean pressure gradients of 104 and 66 mmHg, respectively. The mitral valve was also thickened, calcified, and severely stenosed with a valve area of 1.3 cm^2^ by planimetry and 1.4 cm^2^ by pressure half-time with peak and mean pressure gradients of 22 and 12 mmHg, respectively. Estimated pulmonary artery systolic pressures were 60 mmHg. Cardiology was consulted, and they advised surgical replacement of both valves after pregnancy. The patient was also started on metoprolol 25 mg twice daily.

On subsequent follow-up visits, our patient was diagnosed with IUGR and thus planned for an early cesarean section at 34 weeks of gestation. Cardiology, cardiothoracic surgery, and cardiac anesthesia teams were taken on board. After a thorough discussion, the patient was planned for LSCS under general anesthesia. A baseline blood workup was sent, which was within normal limits.

On the morning of the surgery, the patient was premedicated with 3.75 mg of midazolam (per oral) to alleviate anxiety and avoid stress-induced hemodynamic changes. In the preoperative area, the patient was nebulized with 4% lidocaine 3 cm^3^ in 2 cm^3^ normal saline for 15 min to minimize stress response associated with laryngoscopy and tracheal intubation. Monitoring in the operating room included noninvasive blood pressure (BP), pulse oximetry, and ECG with CM5 configuration. Defibrillator pads were also attached at the back and connected to a defibrillator. Baseline readings showed a BP of 102/65 mmHg, a pulse of 65/min, and 98% room air oxygen saturation. The patient was kept in a left tilt position to minimize aortocaval compression. Preinduction, two wide bore I/V cannulas (B.Braun, Vasofix Safety 16G and 18G) and arterial line (Vygon Arterial Leadercath 3 Fr, 8 cm) were placed. The patient was then preoxygenated for 3 min using a tight-fitting facemask. During this time, the abdomen was prepped and draped by the obstetric team, and simultaneously, the chest was prepped and draped by a cardiothoracic surgeon to be prepared for emergency sternotomy and valve replacement. A perfusionist remained on standby along with the heart and lung machine. Intravenous coinduction was performed with ketamine 20 mg and propofol 50 mg along with succinylcholine 100 mg. Endotracheal intubation was performed with McGrath laryngoscope and 7-mm-cuffed endotracheal tube was passed, and the obstetric team was asked to proceed with the surgery. Anesthesia was maintained with sevoflurane 2–3% in an oxygen and air mixture. Cis-atracurium 0.2 mg/kg was used for muscle relaxation after the return of the train of four. Within 3 min, the baby was delivered alive and well with good Apgar. During this time, ultrasound-guided central line (Arrow, Triple Lumen, 7 Fr, 16 cm) was placed in the right internal juglar vein, and norepinephrine (0.02–0.04 μg/kg/min) was used to maintain systolic BP between 100 and 120 mmHg. The baseline central venous pressure was 15 cmH_2_O. Morphine 0.1 mg/kg and paracetamol 15 mg/kg were used for pain management after the baby was delivered, and ondansetron 0.15 mg/kg was used to prevent postoperative nausea and vomiting. Low-dose oxytocin infusion at 1.25 IU/h was started to help maintain uterine tone without compromising hemodynamics. The surgery lasted for 36 min during which time strict hemodynamic control with a target systolic BP of 100–120 mmHg and target heart rate between 60 and 70/min was maintained. Surgeon was asked to infiltrate the wound with 0.25% ropivacaine 20 cm^3^. Norepinephrine was tapered off by the end of the procedure, and the patient was extubated uneventfully.

Postoperatively, the patient was shifted to the coronary care unit for overnight monitoring. She remained stable and was stepped down to the general obstetric ward the next day and discharged 2 days after surgery.

## Discussion

Cardiac diseases are among the leading causes of indirect maternal mortality in the last Confidential Enquiry into Maternal Deaths^1^. Valvular heart disease comprises 80% burden of patients with heart disease during pregnancy in developing countries, with rheumatic fever as the most common etiology. It may present for the first time during pregnancy^4^.

Among valvular disorders, stenotic valvular lesions may not be well tolerated during pregnancy, and delivery carries a greater risk of maternal and fetal morbidity and mortality as compared to regurgitant lesions mainly because stenotic lesions have limited ability to respond to increases in cardiac output^4^. Pregnancy-associated increase in cardiac output increases the transvalvular gradient and upstream pressure, whereas a fall in the systemic vascular resistance (SVR) will further potentiate fluid retention and volume expansion. This phenomenon appears to be more marked in pregnancy with a stenotic lesion because these patients are unable to respond to the pressure drop with an increase in cardiac output^5^. Regurgitant lesions are generally better tolerated, especially if the underlying cardiac function is normal^4^.

Mitral stenosis is the most common valvular disorder of pregnancy in developing countries such as Pakistan^6^. Patients with severe mitral stenosis (valve area <1.0 cm^2^) have an increased risk of complications and are likely to decompensate^4^. The incidence of maternal cardiac complications correlate with the severity of the mitral stenosis (67% for severe, 38% for moderate, and 26% for mild disease)^7^.

Aortic stenosis in pregnancy is rare. Features that predict a favorable outcome during pregnancy include the absence of symptoms, normal ECG, aortic valve area of at least 1 cm^2^, and preserved left ventricular function^4^. Overall maternal mortality is less than 1%; however, the incidence of complications for both mother and fetus increases with increasing severity of stenosis. At least one-third of pregnant females with aortic valve areas less than 1.5 cm^2^ require hospitalization. The risk of fetal morbidities, including fetal growth restriction, respiratory distress, and preterm birth, also correlates with the severity of aortic stenosis^8^. This is in line with our patient's progress as she developed IUGR.

Generally, the risks of mixed valvular lesions depend upon the dominant abnormality. Left-sided cardiac valvular disease is associated with greater risk, and a dominantly stenotic lesion is more likely to develop complications^4^.

Our patient, however, had coexisting severe stenotic lesions of the left side (both mitral and aortic stenosis) and hence posed a clinical dilemma regarding the choice of anesthesia and perioperative management. Literature is scarce regarding the anesthetic management of combined severe mitral and aortic stenosis and is largely conflicted regarding the choice of general or regional anesthesia. After a multidisciplinary meeting that involved obstetrics, cardiology, cardiac surgery, and anesthesiology teams, we decided to proceed with general anesthesia. Our choice for general anesthesia was based on several factors. Regional anesthesia, especially epidural technique, may prove beneficial for labor in low doses as it decreases catecholamine release and hemodynamic stress^9^; however a critical problem with these patients is the inability to maintain cardiac output with a decrease in SVR and the dosage required to achieve surgical anesthesia, even when employing a graded epidural technique may have been enough to cause significant vasodilation^2^. Moreover, coexisting mitral stenosis further decreases cardiac output leading to hypotension-induced myocardial ischemia, and hence epidural-induced vasodilation could have been catastrophic in this patient^2^. Surgical anesthesia with an epidural is sometimes hampered by patchy or partial block, causing significant distress to the patient along with associated hemodynamic changes, which was another reason why we preferred general anesthesia for our patient^10^. Lastly, our patient’s valvular disorder was significant enough to warrant an emergency double valve replacement in case of complications. Keeping in mind the above-mentioned reasons, general anesthesia seemed the most plausible technique for our patient.

The key issue with general anesthesia was the alteration in hemodynamics associated with tracheal intubation^11^. The placement of a preinduction arterial line helped us in monitoring and tightly control our patient’s hemodynamics. Stress response may be obtunded with the use of opioid-based general anesthesia; however, significant concerns exist regarding neonatal respiratory depression^11^. Considering IUGR and preterm delivery, we decided against using opioids at induction. Instead, we relied on a small dose of oral premedication with midazolam and used lidocaine nebulization which prevented significant alteration in our patient’s BP and heart rate at induction. Kadni *et al*. utilized a similar technique to alleviate stress response to intubation in their patient with combined valvular heart disease undergoing laparoscopic distal gastrectomy. They utilized 15% lidocaine oral spray and intravenous midazolam to minimize stress response^12^.

Maintenance of uterine tone can be challenging in patients with combined valvular disease, especially aortic stenosis. Large oxytocin boluses can cause hypotension, tachycardia, and pulmonary artery hypertension. Careful administration of diluted oxytocin is usually better tolerated^2^.

Regardless of the anesthetic technique, anesthetic goals for a patient with the combined valvular disease should remain the same^2^. Maintenance of adequate preload, SVR, cardiac contractility, sinus rhythm, and avoidance of tachycardia, bradycardia, aortocaval compression, and anesthetic or surgery-induced hemodynamic changes comprise the mainstay of perioperative management.

## Ethical approval

Not required.

## Patient consent

Obtained.

## Sources of funding

None.

## Author contribution

H.S.: performed the case, took consent, wrote the report, and reviewed the final version; K.S.: performed the case, edited the report, and reviewed and approved the final version; A.A.: edited the report, wrote the abstract, and reviewed the final version.

## Conflicts of interest disclosure

The authors declare that they have no conflicts of interest.

## Research registration unique identifying number (UIN)

None.

## Guarantor

I, Haris Sheikh, take full responsibility for the work done on this case report and will be the one to correspond with the journal and other correspondence as per need.

## Data availability statement

None.

## Provenance and peer review

Not commissioned, externally peer-reviewed.
